# MRI Pattern Recognition in Multiple Sclerosis Normal-Appearing Brain Areas

**DOI:** 10.1371/journal.pone.0021138

**Published:** 2011-06-17

**Authors:** Martin Weygandt, Kerstin Hackmack, Caspar Pfüller, Judith Bellmann–Strobl, Friedemann Paul, Frauke Zipp, John­–Dylan Haynes

**Affiliations:** 1 Bernstein Center for Computational Neuroscience Berlin, Charité - University Medicine, Berlin, Germany; 2 NeuroCure Clinical Research Center, Charité - University Medicine Berlin, Berlin, Germany; 3 Experimental and Clinical Research Center, Charité - University Medicine Berlin, Berlin, Germany; 4 Clinical and Experimental Multiple Sclerosis Research Center, Charité - University Medicine Berlin, Berlin, Germany; 5 Department of Neurology, University Medicine Mainz, Johannes Gutenberg University, Mainz, Germany; 6 Max­Planck­Institute for Human Cognitive and Brain Sciences, Leipzig, Germany; Julius-Maximilians-Universität Würzburg, Germany

## Abstract

**Objective:**

Here, we use pattern-classification to investigate diagnostic information for multiple sclerosis (MS; relapsing­remitting type) in lesioned areas, areas of normal­appearing grey matter (NAGM), and normal-appearing white matter (NAWM) as measured by standard MR techniques.

**Methods:**

A lesion mapping was carried out by an experienced neurologist for Turbo Inversion Recovery Magnitude (TIRM) images of individual subjects. Combining this mapping with templates from a neuroanatomic atlas, the TIRM images were segmented into three areas of homogenous tissue types (Lesions, NAGM, and NAWM) after spatial standardization. For each area, a linear Support Vector Machine algorithm was used in multiple local classification analyses to determine the diagnostic accuracy in separating MS patients from healthy controls based on voxel tissue intensity patterns extracted from small spherical subregions of these larger areas. To control for covariates, we also excluded group-specific biases in deformation fields as a potential source of information.

**Results:**

Among regions containing lesions a posterior parietal WM area was maximally informative about the clinical status (96% accuracy, p<10^−13^). Cerebellar regions were maximally informative among NAGM areas (84% accuracy, p<10^−7^). A posterior brain region was maximally informative among NAWM areas (91% accuracy, p<10^−10^).

**Interpretation:**

We identified regions indicating MS in lesioned, but also NAGM, and NAWM areas. This complements the current perception that standard MR techniques mainly capture macroscopic tissue variations due to focal lesion processes. Compared to current diagnostic guidelines for MS that define areas of diagnostic information with moderate spatial specificity, we identified hotspots of MS associated tissue alterations with high specificity defined on a millimeter scale.

## Introduction

Following the current diagnostic guidelines for multiple sclerosis (MS), the so­called ‘McDonald criteria’ [1], [2] and novel criteria [3], exclusively lesion related MR criteria are considered diagnostically informative. However, recent studies using conventional MRI as well as more advanced imaging sequences have identified additional markers that were abnormal in MS patients. For example, recent studies found evidence for grey matter (GM) volume loss in MS patients [4], [5]. These GM alterations could be detected using conventional MR sequences. However, there is also a variety of disease­related features that could be detected only with advanced MR techniques (e.g. magnetic resonance spectroscopy [6], [7]; diffusion tensor imaging [8], [9]; high­field MRI [10], [11]) and appeared to be ‘invisible’ in standard MR images. Due to this dissociation, such areas have been termed areas of ‘normal­appearing white matter’ (NAWM) and ‘normal­appearing grey matter’ (NAGM) respectively [12]. Collectively, they are known as normal-appearing brain tissue (NABT; [13]).

Recent work applying pattern recognition techniques (‘classifiers’) to the analysis of neuroimaging data could show that these methods have higher sensitivity than traditional approaches, i.e. conventional statistical methods [14] and visual inspection [15]. These studies demonstrated that classifiers were more successful than conventional statistical approaches in detecting cognitive states from fMRI data in healthy subjects [16], [17], [18] as well as human diagnosticians in detecting Alzheimer's disease based on structural MR images [14], [15]. Please see [14] for an overview on pattern recognition in MRI. Finally, classifiers have been used to detect neurological diseases based on high-resolution MR images [15], [19], [20], [21], however not yet in MS.

In the present study, we investigate whether MS patients can be separated from healthy controls acquired by conventional MR using pattern classification strategies. We conducted three analyses based on structural T2­weighted images. In the lesion area analysis, we searched across all brain areas containing hyperintense lesioned tissue for regional intensity patterns that are informative about the clinical condition. In the normal-appearing grey matter area analysis and the normal-appearing white matter area analysis, we analyzed brain areas that exclusively contained normal­appearing brain tissue. Using this approach, we could investigate whether classifiers can extract diagnostic information for MS from regions that appear normal in conventional MRI.

## Materials and Methods

### Participants

Forty-one patients with MS (relapsing­remitting type; [1]) and twenty-six age and gender matched healthy controls participated in the study (mean age patients  = 35.7±7.4 years; mean age controls  = 38.7±11.6 years; 21 female and 20 male patients; 14 female and 12 male controls). Patients exhibited a mild to moderate symptom severity as indicated by the Expanded Disability Status Scale (median  = 2.0, range  = 0.0–7.0). Mean disease duration of patients was 80.0 (±76.3) month.

### Ethics statement

Consent was obtained according to the Declaration of Helsinki, and the study was approved by the research ethics committee of the Charité - University Medicine Berlin. All subjects gave written informed consent.

### Brain imaging

Whole brain high resolution 3­dimensional T1­weighted images (MPRAGE, TR 2110 ms, TE 4.38 ms, TI 1100 ms, flip angle 15°, resolution 0.5×0.5×1 mm, axial acquisition direction) and T2­weighted images (TIRM, TR 10000 ms, TE 108 ms, TI 2500 ms, resolution 0.5×0.5×3 mm, 44 contiguous axial slices) were acquired using a 1.5 Tesla whole­body tomograph (Magnetom Sonata, Siemens, Erlangen, Germany) with a standard head coil.

### Data preprocessing

A clinician (CP) used in-house software to conduct a lesion mapping based on subjects' native TIRM images having a voxel resolution of 0.5×0.5×3 mm. Correction of field­inhomogeneities, within­subject image coregistration of TIRM images to high resolution MPRAGE images and spatial normalization of high resolution images to the Montreal Neurological Institute (MNI) brain template (voxel resolution: 2×2×2 mm) were performed using SPM5 (Wellcome Trust Centre for Neuroimaging, Institute of Neurology, UCL, London UK ­ http://www.fil.ion.ucl.ac.uk/spm). Individual lesion areas were excluded in the normalization routine to avoid lesion-mediated deformation artefacts. Additionally, they were used to generate a group lesion mask (brain locations where lesions occurred in at least one across all subjects). The group lesion mask was then combined with templates for grey and white matter taken from the neuroanatomic WFU pickatlas [22] to generate a group NAGM mask (coordinates that exclusively contained NAGM across the sample), a group NAWM mask, and a group NABT mask defined in the space of the MNI brain template. Then, we conducted within-subject image intensity standardization. Data resulting from these steps are referred to as ‘uncorrected data’ as they were not corrected for deformation confounds (see below).

**Figure 1:**
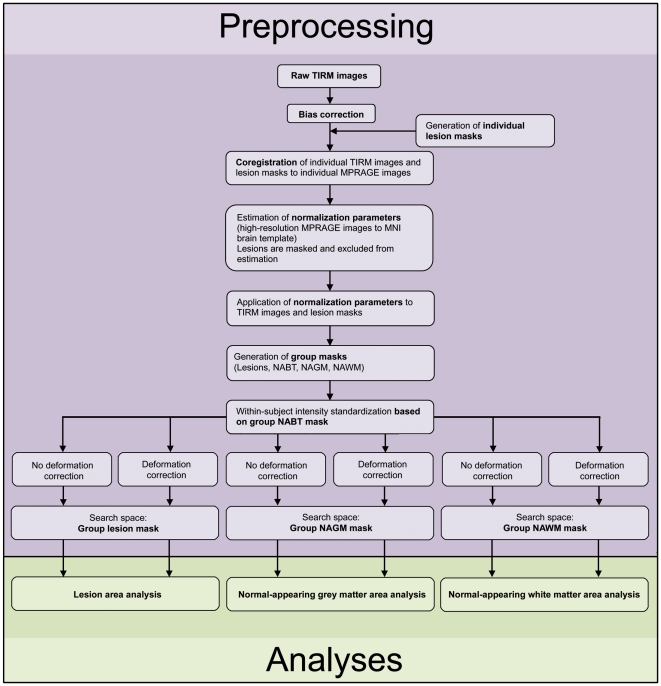
Overview of data processing. For details please see text and Material S1. MNI, Montreal Neurological Institute; NAGM, normal­appearing grey matter; NAWM, normal-appearing white matter; NABT, normal-appearing brain tissue.

We continued by regressing out the variance contained in tissue intensities that could be explained by the local deformation parameters determined during spatial normalization. This step was performed to rule out that classification could rely on systematic intensity differences between groups induced by the spatial transformation (e.g. due to correction of thalamic atrophy in patients only). These data are referred to as ‘corrected data’. Then, we conducted between-subject z-transformation of the corrected and uncorrected data to account for intensity variations e.g. due to coil loadings. Finally, corrected and uncorrected data (voxel resolution: 2×2×2 mm) were restricted to the search space of each analysis defined by the group masks described above and entered the analyses. See Figure 1 and Material S1 for further details.

### Pattern recognition analyses

We assessed whether small brain areas located either in lesioned tissue, normal-appearing grey matter tissue, or normal-appearing white matter tissue contain diagnostically relevant information about the clinical status (‘MS’ or ‘healthy control’). For that we conducted three tissue specific pattern recognition analyses (lesion area analysis, NAGM area analysis, and NAWM area analysis) using in-house software [23]. Each analysis was once based on uncorrected data and once on data corrected for deformation confounds.

**Figure 2:**
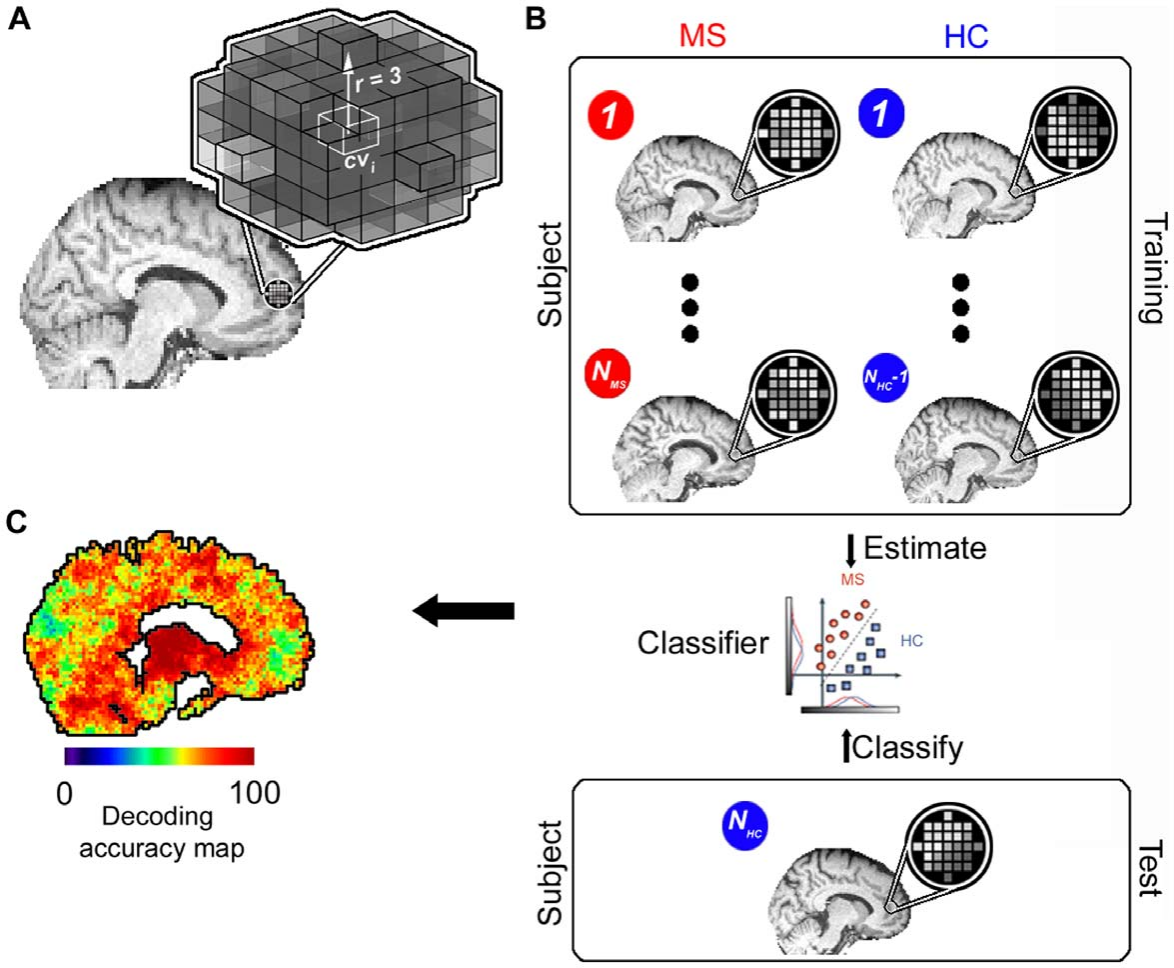
Mapping of brain regions with diagnostic information. (A) ‘Searchlight’ approach that searches across the brain for local tissue intensity patterns that are informative about the clinical condition (MS, healthy control [HC]). For a given ‘center’ voxel cv_i_ in the brain the searchlight is defined as a spherical cluster with a radius of three voxels surrounding the center coordinate. Thus, a searchlight contained 123 spherically arranged voxels if the minimal distance to the boundary of the search space was at least three voxels. However, when the center voxel was located closer to a boundary of the search space of a given analysis the searchlight could deviate from the spherical shape and contain less voxels in order to guarantee that only voxels belonging to the supposed tissue class were contained in searchlights in a given analysis. (B) Within this cluster of voxels the spatial pattern of intensities is extracted for each subject separately. The data from all (N_total_  =  N_MS_ + N_HC_) but one subject (N_total_­1) are used as a ‘training dataset’ to train a classifier to distinguish between patterns from the two groups. The classifier is then tested by applying it to the data from the remaining ‘test’ subject (in this example N_HC_). This leave­one­out (LOO) cross­validation procedure was then repeated n­times by leaving out the data of one subject at a time from the training data set. The success of the classifier is an estimate of the local information at that position in the brain. (C) The resulting accuracy was then noted at the coordinate cv_i_ as the local information related to the clinical condition. By iterating this procedure across different positions in the brain it is possible to obtain a map of diagnostic accuracies for each coordinate cv_i_, depicting the diagnostic information contained in local patterns in decoding the clinical condition.

To identify diagnostically informative areas we used a searchlight approach [17], [24] that determines the separability of MRI intensity patterns of patients and controls for small spherical brain areas aligned on a center voxel with a radius of three voxels. In each tissue specific analysis each voxel was once treated as center voxel of a searchlight and the separability of patterns of both groups extracted from this spherical area was determined by a pattern classifier. The procedure resulted in a map of classification accuracies depicting the local diagnostic information for the discrimination of clinical groups for the given tissue class. For individual searchlights, we extracted the TIRM image voxel intensities from the spatially standardized and otherwise preprocessed images (see above) of all (N) subjects separately in a first step. This intensity information was then recorded in a data matrix where each voxel of the searchlight was represented by a column and each subject by a row. Leave­one­out (LOO) cross­validation was used to determine the success of the classifier in separating between searchlight tissue intensity patterns of patients and controls. In this procedure, patterns from N-1 subjects (N-1 rows of the data matrix) were fed into the classifier as a ‘training dataset’. The classifier then determined a linear decision boundary to separate between training patterns of patients and controls. Next, the classifier was tested by applying the decision boundary to the data from the remaining, independent ‘test’ subject (recorded in the row of the data matrix not used for training). The procedure was repeated N times until the clinical status of each subject was predicted based on the corresponding intensity pattern / until each pattern was left out once from the training dataset. Finally, the mean of sensitivity and specificity was calculated for this searchlight classifier and noted at the center voxel of the searchlight in the accuracy map as a local measure of diagnostic information. We preferred this accuracy measure over the percentage of true classification decisions as the mean of sensitivity and specificity accounts for the unbalanced number of subjects in the two groups. For further details, see Figure 2.

The classification algorithm we used was a linear Support Vector Machine (SVM) classifier [25] and is available at http://www.cs.wisc.edu/dmi/svm/nsvm/nsvm.m. The algorithm attempts to find a linear decision boundary that separates the patterns of two classes (here: local searchlight tissue intensity patterns of MS patients and healthy controls respectively). During identification of the decision boundary the algorithm optimizes a free parameter that determines the tradeoff between classifier complexity and number of non-separable patterns (please see [26] for further details). Instead of explicitly using cross-validation to optimize this parameter, the algorithm approximates the cross-validation rate as this procedure saves computational cost.

**Figure 3:**
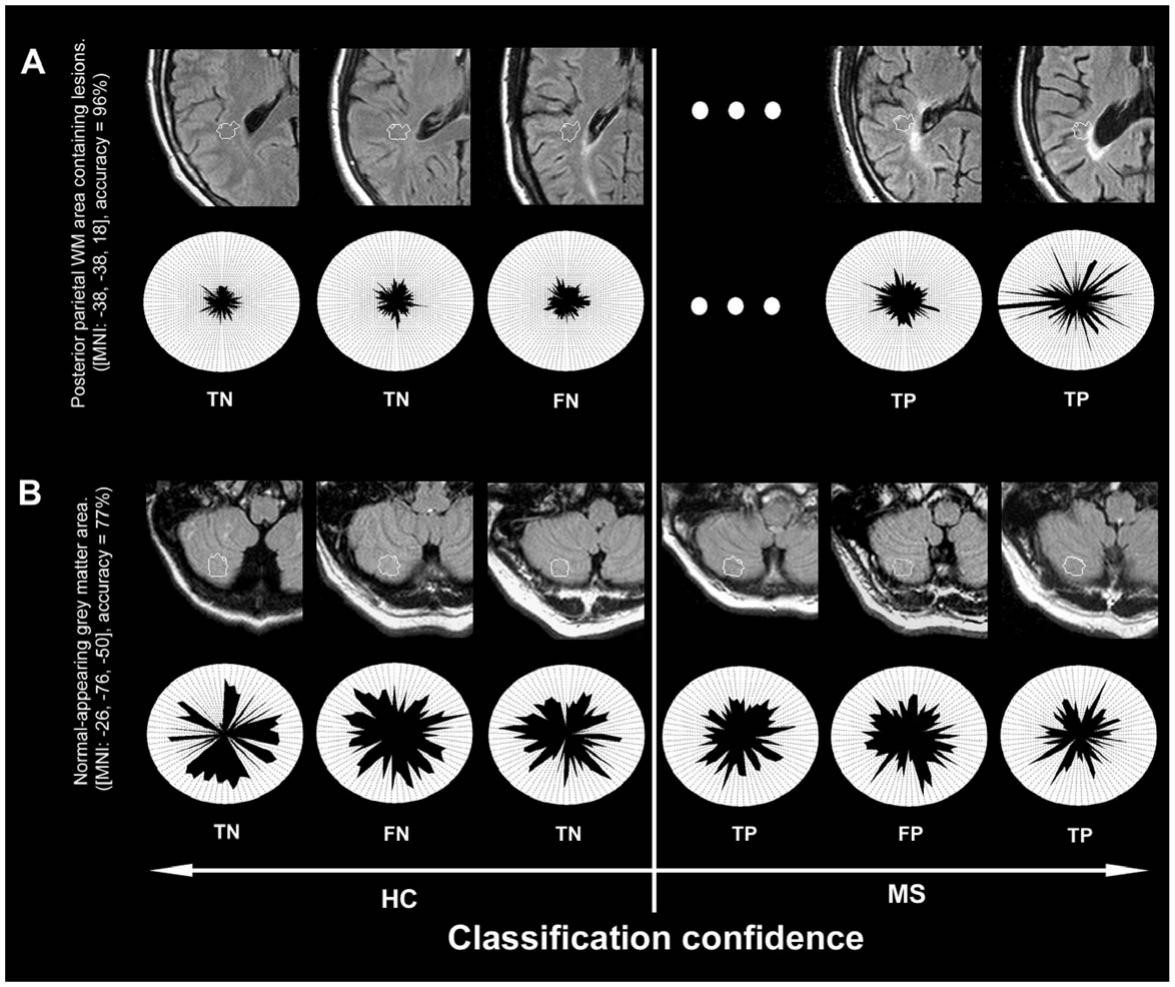
Image features with information on the clinical status. In order to provide insights into pathology indicating MR image features we depict exemplary searchlight classifiers in the native space of individual subjects, i.e. their native TIRM images (0.5×0.5×3 mm voxel resolution) and highlight the pattern structure of selected subjects. (A) Posterior parietal white matter searchlight that obtained maximal accuracy in the lesion area analysis for uncorrected data (96%, p<10^­13^). Top row: Outer contour lines correspond to the border of searchlights. Inner contour lines correspond to the voxel in the respective searchlight that was most important for the multivariate decision process. The sorting of individual images from left to right follows the diagnoses of the classifiers (left: images that were diagnosed as controls, right; images that were diagnosed as patients; eccentricity follows diagnostic confidence). Bottom row: Polar plot of the tissue intensity patterns drawn from the voxels located in this area in the normalized TIRM images of individual subjects (2×2×2 mm voxel resolution), sorted clockwise by the relevance of a voxel for the classification in descending order. The plot suggests that the classifier grounds its decisions mainly on a set of hyperintense voxels in patients that are distributed across the searchlight. (B) Searchlight of maximal accuracy in the NAGM area analysis based on data corrected for deformation effects. The TIRM image characteristics and the polar plot depicted underline the capability of the pattern recognition approach to identify disease indicating information from brain tissue characteristics ‘invisible’ to the human eye. TP, true positive; TN, true negative; FP, false positive; FN, false negative.

Probabilities of observed classification accuracies were calculated using the χ2 ­ distribution. We applied different p-thresholds for the analyses to guarantee clarity and comprehensibility of the results presentation. For the lesion area analysis based on uncorrected as well as corrected data and for NAGM and NAWM area analysis based on uncorrected data we report searchlight center coordinates cv_i_ that exhibit a significant accuracy on a family­wise­error (FWE; Bonferroni correction) corrected level p_FWE_<0.05. For the lesion area analysis based on uncorrected data, we additionally defined a cluster size criterion for significant coordinates to maximize the specificity of the analysis, i.e. a number (k) of neighbouring searchlights yielding significant accuracies and set this threshold to k = 5. For the NAGM and NAWM area analysis based on corrected data, we report coordinates cv_i_ that exhibit an accuracy on a FWE-corrected level of p_FWE_<0.1 and coordinates that exhibit a trend towards significance (p_uncorrected_  = 0.001). To provide insights into the image features and the neuropathology underlying local separability of groups, we computed a conventional t­contrast between patients and controls based on the TIRM images of each subject for each voxel underlying a cluster of above­chance searchlight classifier. We assume that high positive t­values are suggestive of tissue damage due to e.g. demyelination, oedema, and inflammation [27], whereas pronounced negative t­values may be suggestive of tissue atrophy e.g. based on iron deposition [28]. This contrast can also clarify whether multivariate separability is driven by pronounced voxel­wise intensity differences or subtle interactions among variables. Results report the percentage of voxels located within the radius of (a cluster of) significant searchlights that showed a significant intensity difference between groups (p_uncorrected_  = 0.001, no cluster size criterion, two­sided) and the mean t­values for these voxels. Furthermore, we depict native TIRM images of individual subjects for selected coordinates in order to illustrate image features with information on the clinical status (Figure 3).

## Results

### Lesion area analysis

**Figure 4:**
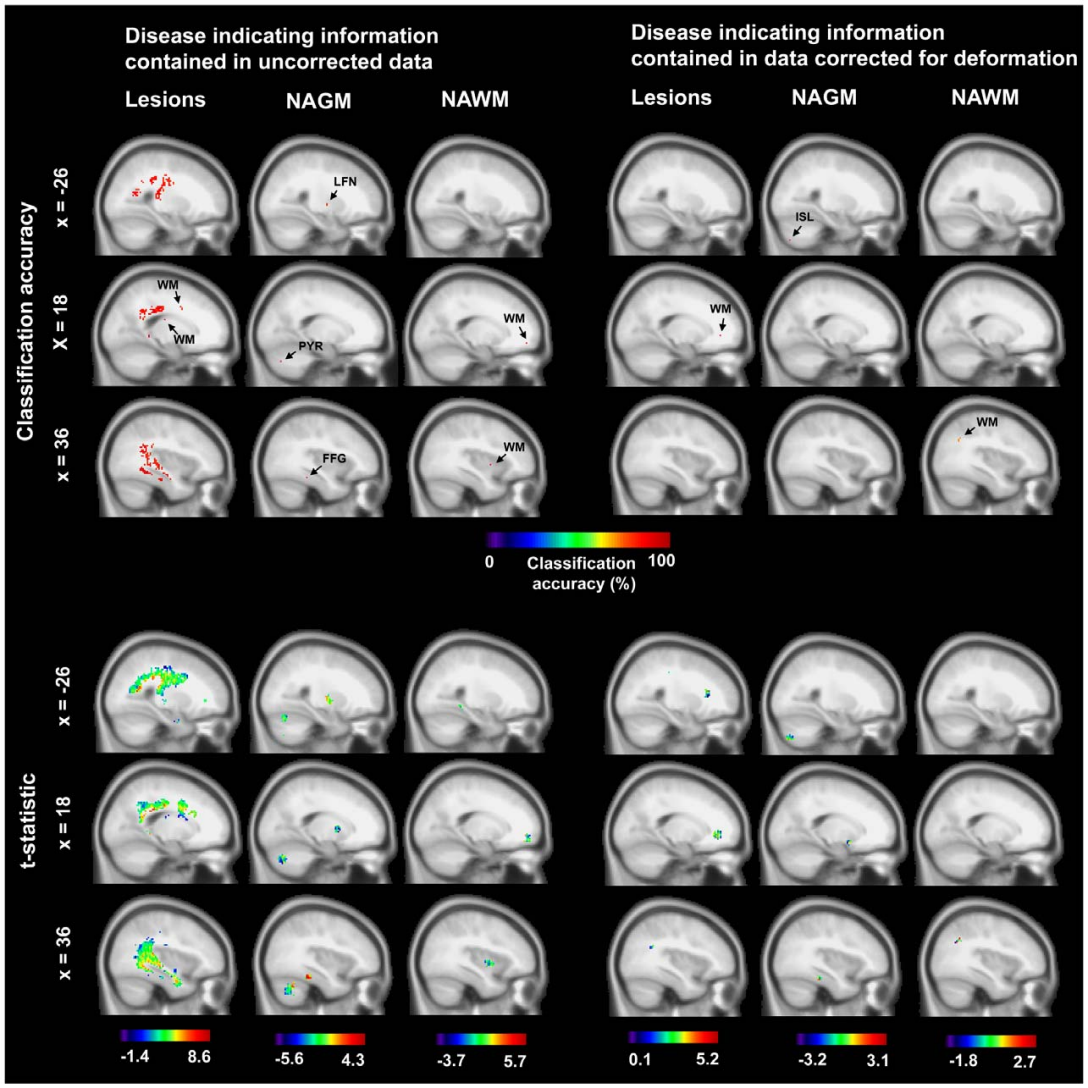
Brain regions with information on the clinical status. Top: Center coordinates of searchlight classifiers with above­chance accuracy in the separation of patients and controls. Bottom: Tissue intensity differences between patients and controls for voxels underlying significant searchlight classifiers (t­statistic; MS minus control). Left: Analysis based on raw data. Right: Analysis is based on data corrected for deformation. FFG, fusiform gyrus; ISL, inferior semi-lunar lobule; LFN, lentiform nucleus; PYR, pyramis; WM, white matter.

For uncorrected data maximal accuracy for the separation of patients and controls was obtained in a posterior parietal WM area ([MNI: ­38, ­38, 18], accuracy  = 96%, p<10^­13^, corr., significant voxels  = 53%, mean t­value  = 3.6). Interestingly, only 19 of 41 patients had at least one lesion in the corresponding searchlight. Thus, a maximal accuracy (i.e. mean of sensitivity and specificity) of 73% could have been obtained given that controls do not have lesions and classification relies exclusively on lesions. Consequently, accuracy could impossibly rely on the presence of lesions alone. Figure 3 shows native TIRM image features used by pattern classifiers for this coordinate. In addition to white matter structures the caudate nucleus also contained significant information on the clinical status ([MNI: −34, −16, −12], accuracy  = 86%, p<10^−7^, corr., significant voxels  = 83%, mean t­value  = 4.5). On average across all areas obtaining significant accuracies the contribution of significant tissue intensity differences between patients and controls on the voxel level for separation was very strong (55±14%). See Material S1, Table S1 and Figure 4 for further details.

For deformation corrected data, a frontal WM area achieved the highest accuracy in separating between patients and controls ([MNI: 22, 38, −2], accuracy  = 83%, p<10^−6^, corr., significant voxels  = 22%, mean t-value  = 2.9). 13 out of 41 patients had a lesion in that area. Correspondingly, a maximal accuracy of 66% could have been obtained given that controls do not have lesions and classification relies exclusively on lesions. The contribution of significant tissue differences between patients and controls on the voxel level for the separation of groups was much smaller as compared to the analysis based on uncorrected data. On average, 20% (SD  = 19%) of all voxels involved showed a significant tissue intensity difference. See Table S2 and Figure 4 for further details.

### Normal-appearing grey matter area analysis

Predominantly cerebellar regions and deep grey matter nuclei were informative about the clinical condition for data not corrected for deformation effects. The highest accuracy in cerebellar regions was obtained in the declive ([MNI: 2, −22, −70], accuracy  = 84%, p<10^−7^, corr., significant voxels  = 1%, mean t­value  = −1.5). Among deep grey matter nuclei, the highest accuracy was obtained for regions in the lentiform nucleus ([MNI: 3, −26, 2], accuracy  = 82%, p<10^−6^, corr., significant voxels  = 0%, mean t­value  = −0.6). As is indicated by mean t-values these areas were mainly characterised by hypointense signals in patients which is suggestive of brain atrophy. However, although this pattern of hypointensity was quite consistent across regions the difference was not very pronounced. On average across all voxels involved, only 5% (SD  = 6%) showed a significant difference. See Table S1 and Figure 4.

Among data corrected for deformation confounds, maximal accuracy was obtained in the inferior semi-lunar lobule of the cerebellum ([MNI: −26, −76, −50], accuracy  = 77%, p<10^−5^, corr., significant voxels  = 0%, mean t-value  = −0.3). Moreover, a parahippocampal area obtained significant results ([MNI: 38, −30, −18], accuracy  = 70%, p <10^−3^, uncorr., significant voxels  = 0%, mean t-value  = 0.6). Finally, high accuracy was also obtained for a region in the subcalossal gyrus ([MNI: 16, 4, −14), accuracy  = 70%, p <10^−3^, uncorr., significant voxels  = 0%, mean t-value  = −0.7). Pronounced intensity differences on the voxel level did not contribute to the separation of patients and controls at all. See Table S2 and Figure 4 for full details. See Figure 3 for image features used by pattern classifiers in the inferior semi-lunar lobule.

### Normal-appearing white matter area analysis

For uncorrected data maximal accuracy for the separation of MS patients and controls was obtained in a posterior NAWM area ([MNI: −22, −42, −6), accuracy  = 91%, p<10^−10^, corr., significant voxels  = 17%, mean t­value  = 1.6). NAWM areas separating between groups were predominantly characterized by hyperintense signals in patients. The contribution of significant tissue differences between patients and controls on the voxel level for separation was relatively weak. On average, 16% (SD  = 20%) of all voxels involved showed a significant tissue intensity difference. See Table S1 and Figure 4.

Importantly, we also found significant results when we corrected for deformation effects in two NAWM areas ([MNI: 36, −60, 30], accuracy  = 71%, p<10^−3^, uncorr., significant voxels  = 0%, mean t-value  = 0.6; ([MNI: −56, −12, 30], accuracy  = 71%, p<10^−3^, uncorr., significant voxels  = 0%, mean t-value  = 1.1). Significant tissue intensity differences on the voxel level did not contribute to the separation of groups at all. See Table S2 and Figure 4.

## Discussion

The present study demonstrates that MS patients can be separated from healthy controls based on local brain tissue intensity patterns by a combination of conventional MR techniques and pattern recognition algorithms. Among regions containing lesions, especially posterior parietal WM areas were informative about the clinical status of subjects. When classification relied exclusively on NABT, especially deep GM nuclei and cerebellar areas contained information.

We performed three pattern classification analyses based on T2­weighted images following a lesion­mapping conducted by an experienced neurologist: A lesion area analysis, a NAGM area analysis, and a NAWM area analysis. Each analysis was once based on uncorrected data and once on data corrected for deformation confounds.

In the lesion area analysis, we investigated whether patients can be separated from controls based on regional tissue intensity patterns containing macroscopic lesions. This allowed evaluating the variation of disease indicating information of lesions depending on their precise location defined on the millimeter scale. For data not corrected for deformation maximal accuracy was obtained in a posterior parietal WM area (accuracy  = 96%). However, also deep gray matter structures obtained a high accuracy (caudate nucleus, accuracy  = 86%). For corrected data, a frontal WM area turned out to be maximally informative (accuracy  = 83%). In order to clarify to which extent separation could be driven exclusively by the presence of lesions, we determined the number of patients that had at least one lesion in the areas of maximal separation for deformation corrected and uncorrected data. It turned out that the accuracy obtained empirically was much higher than would have been possible when classification would have relied exclusively on lesions. This indicates that classifiers extracted diagnostic information from two sources: macroscopically visible lesions and subtle signal variations. This suggests that classifiers captured the outcome of a continuous disease process ranging from normal tissue in controls over NABT to lesions as an endpoint in patients.

In the NAGM area analyses, especially deep GM nuclei and cerebellar brain regions were identified as informative regions. Maximal accuracy was obtained in cerebellar regions (uncorrected data: 84% accuracy; deformation corrected data: 77% accuracy). Nearly all informative areas were on average characterised by slightly decreased signals in patients. Hypointensity of deep GM nuclei in T2­weighted images has been demonstrated in recent studies [29]. It has been linked to brain atrophy resulting from elevated iron deposition in MS [28]. Future studies are necessary to investigate the relationship of informative NABT areas revealed by pattern classifiers in standard MR images and metabolic alterations in these areas measured by MRS or alterations in diffusion processes measured by DTI. The contribution of significant intensity differences in individual voxels to the separation of groups was small. This suggests that multivariate classifiers did not mainly rely on information from voxels with strong intensity differences, but included information from voxels that show weak differential effects. These results and findings as the cerebellar image features depicted in Figure 3 suggest that pattern recognition methods can extract diagnostic information from subtle interactions between brain tissue intensity variations that are hard to detect for the visual system.

Among NAWM areas a posterior region obtained maximal accuracy (accuracy  = 91%). Most of the informative NAWM areas were characterized by slight hyperintensity in patients, although hyperintensity was much less pronounced than for lesions. This matches the criteria of so-called ‘dirty-appearing’ white matter that has higher intensity as normal WM but lower intensity than lesions [30]. However, additional studies are needed investigating the neuropathological foundations of informative NAWM areas as identified in this study.

To summarize, we identified regions with information for MS in lesioned, but also NAGM, and NAWM areas. This proves that standard MR techniques have sufficient sensitivity to capture fine-grained tissue alterations in normal-appearing brain areas of MS-patients. Furthermore, we obtained a high spatial specificity in detecting brain regions with diagnostic information, as we identified hotspots of MS associated tissue alterations defined on a millimeter scale.

## Supporting Information

Table S1 **Cross-validation results for the mapping of regions with disease indicating information based on uncorrected data. H**, hemisphere; **CS**, cluster size, i.e. the number of neighboring significant searchlights; **x, y, z**, Montreal Neurological Institute coordinate of the center of the searchlight classifier with the peak accuracy; **DA(%)**, decoding accuracy; **p**, probability of the accuracy according to χ2-distribution. **Mn t,** mean t-value for the group contrast patients minus controls for voxels underlying a (cluster of) significant searchlight classifier(s); Vox*(%), percentage of these voxels showing significant results for the contrast (p = 0.001, uncorrected, no cluster size criterion, two-sided).
(DOC)

Table S2 **Cross-validation results for the mapping of regions with disease indicating information based on data corrected for deformation. H**, hemisphere; **CS**, cluster size, i.e. the number of neighboring significant searchlights; **x, y, z**, Montreal Neurological Institute coordinate of the center of the searchlight classifier with the peak accuracy; **DA(%)**, decoding accuracy; **p**, probability of the accuracy according to χ2-distribution. **Mn t**, mean t-value for the group contrast patients minus controls for voxels underlying (a cluster of) significant searchlight classifiers; Vox*(%), percentage of these voxels showing significant results for the contrast (p = 0.001, uncorrected, no cluster size criterion, two-sided).
(DOC)

Material S1 **Material S1 gives further information regarding data preprocessing and detailed results for each tissue-specific pattern recognition analysis (Lesions, NAGM, and NAWM).**
(DOC)

## References

[1] McDonald WI, Compston A, Edan G, Goodkin D, Hartung HP (2001). Recommended diagnostic criteria for multiple sclerosis: guidelines from the International Panel on the diagnosis of multiple sclerosis.. Ann Neurol.

[2] Polman CH, Wolinsky JS, Reingold SC (2005). Multiple sclerosis diagnostic criteria: three years later.. Mult Scler.

[3] Montalban X, Tintoré M, Swanton J, Barkhof F, Fazekas F (2010). MRI criteria for MS in patients with clinically isolated syndromes.. Neurology.

[4] Chard DT, Griffin CM, Rashid W, Davies GR, Altmann DR (2004). Progressive grey matter atrophy in clinically early relapsing-remitting multiple sclerosis.. Mult Scler.

[5] Valsasina P, Benedetti B, Rovaris M, Sormani MP, Comi G (2005). Evidence for progressive gray matter loss in patients with relapsing­remitting MS.. Neurology.

[6] Chard DT, Griffin CM, McLean MA, Kapeller P, Kapoor R (2002). Brain metabolite changes in cortical grey and normal­appearing white matter in clinically early relapsing­remitting multiple sclerosis.. Brain.

[7] Inglese M, Ge Y, Filippi M, Falini A, Grossman RI (2004). Indirect evidence for early widespread gray matter involvement in relapsing-remitting multiple sclerosis.. NeuroImage.

[8] Roosendaal SD, Geurts JJG, Vrenken H, Hulst HE, Cover KS (2009). Regional DTI differences in multiple sclerosis patients.. NeuroImage.

[9] Vrenken H, Pouwels PJ, Geurts JJG, Knol DL, Polman CH (2006). Altered diffusion tensor in multiple sclerosis normal­appearing brain tissue: cortical diffusion changes seem related to clinical deterioration.. J Magn Reson Imaging.

[10] Schmierer K, Parkes HG, So PW, An SF, Brandner S (2010). High field (9.4 Tesla) magnetic resonance imaging of cortical grey matter lesions in multiple sclerosis.. Brain.

[11] Mainero C, van der Kouwe A, Benner T, Radding A, Jensen R (2009). In vivo imaging of cortical pathology in multiple sclerosis using ultra­high field MRI.. Neurology.

[12] Filippi M, Comi G, Rovaris M (2004). Normal­appearing White and Grey Matter Damage in Multiple Sclerosis..

[13] Filippi M, Tortorella C, Rovaris M, Bozzali M, Possa F (2000). Changes in the normal appearing brain tissue and cognitive impairment in multiple sclerosis.. J Neurol Neurosurg Psychiatry.

[14] Haynes JD, Rees G (2006). Decoding mental states from brain activity in humans.. Nat Rev Neurosci.

[15] Kloeppel S, Stonnington CM, Barnes J, Chen F, Chu C (2008). Accuracy of dementia diagnostic - a direct comparison between radiologists and a computerized method.. Brain.

[16] Cox DD, Savoy RL (2003). Functional magnetic resonance imaging (fMRI) “brain reading”: detecting and classifying distributed patterns of fMRI activity in human visual cortex.. NeuroImage.

[17] Haynes JD, Sakai K, Rees G, Gilbert S, Frith C (2007). Reading hidden intentions in the human brain.. Curr Biol.

[18] Kriegeskorte N, Formisano E, Sorger B, Goebel R (2007). Individual faces elicit distinct response patterns in human anterior temporal cortex.. Proc Natl Acad Sci.

[19] Davatzikos C, Resnick SM, Wu X, Parmpi P, Clark CM (2008). Individual Patient Diagnosis of AD and FTD via High­Dimensional Pattern Classification of MRI.. NeuroImage.

[20] Magnin B, Mesrob L, Kinkingnéhun S, Pélégrini-Issac M, Colliot O (2009). Support vector machine­based classification of Alzheimer's disease from whole­brain anatomical MRI.. Neuroradiology.

[21] Schmah T, Yourganov G, Zemel RS, Hinton GE, Small SL (2010). Comparing Classification Methods for Longitudinal fMRI Studies.. Neural Computation.

[22] Maldjian JA, Laurienti PJ, Burdette JB, Kraft RA (2003). An Automated Method for Neuroanatomic and Cytoarchitectonic Atlas-based Interrogation of fMRI Data Sets.. NeuroImage.

[23] Weygandt M, Schäfer A, Schienle A, Haynes JD (2011). Diagnosing different binge-eating disorders based on reward-related brain activation patterns.. Hum Brain Mapp.

[24] Kriegeskorte N, Goebel R, Bandettini P (2006). Information­based functional brain mapping.. Proc Natl Acad Sci USA.

[25] Fung G, Mangasarian OL (2003). Finite Newton Method for Lagrangian Support Vector Machine Classification. Data Mining Institute.. Technical Report 02­01, Neurocomputing.

[26] Schölkopf B, Smola A (2002). Learning with Kernels..

[27] De Groot CJA, Bergers E, Kamphorst W, Ravid R, Polman CH (2001). Post­mortem MRI­guided sampling of multiple sclerosis brain lesions. Increased yield of active demyelinating and (p)reactive lesions.. Brain.

[28] Ceccarelli A, Filippi M, Neema M, Arora A, Valsasina P (2009). T2 hypointensity in the deep gray matter of patients with benign multiple sclerosis.. Mult Scler.

[29] Bakshi R, Benedict RHB, Bermel RA, Caruthers SD, Puli SR (2002). T2 Hypointensity in the Deep Gray Matter of Patients With Multiple Sclerosis: A Quantitative Magnetic Resonance Imaging Study.. Arch Neurol.

[30] Ge Y, Grossman RI, Babb JS, Juan He, Mannon LJ (2003). Dirty-Appearing White Matter in Multiple Sclerosis: Volumetric MR Imaging and Magnetization Transfer Ratio Histogram Analysis.. Am J Neuroradiol.

