# Supplementary material for: MRI Pattern Recognition in Multiple Sclerosis Normal-Appearing Brain Areas
Source: PLoS One. 2011 Jun 17;6(6):e21138. doi: 10.1371/journal.pone.0021138 (PMC3117878; doi:10.1371/journal.pone.0021138)
Supplement: Table S1 — Cross-validation results for the mapping of regions with disease indicating information based on uncorrected data. H, hemisphere; CS, cluster size, i.e. the number of neighboring significant searchlights; x, y, z, Montreal Neurological Institute coordinate of the center of the searchlight classifier with the peak accuracy; DA(%), decoding accuracy; p, probability of the accuracy according to χ2-distribution. Mn t, mean t-value for the group contrast patients minus controls for voxels underlying a (cluster of) significant searchlight classifier(s); Vox*(%), percentage of these voxels showing significant results for the contrast (p = 0.001, uncorrected, no cluster size criterion, two-sided). (DOC) [file pone.0021138.s001.doc]

**Table S1.** Cross-validation results for the mapping of regions with disease indicating information based on uncorrected data.

| **Tissue / Region** | **H** | **CS** | **x** | **y** | **z** | **DA (%)** | **p** | **Mn t** | **Vox* (%)** |
| --- | --- | --- | --- | --- | --- | --- | --- | --- | --- |
| ***Lesions*** |  |  |  |  |  |  |  |  |  |
| **White matter** | **L** | **1405** | **-38** | **-38** | **18** | **96** | **<10^-13^** | **3.6** | **53** |
|  | **R** | **1059** | **34** | **-52** | **-6** | **94** | **<10^-12^** | **3.7** | **57** |
|  |  | **36** | **20** | **-48** | **24** | **91** | **<10^-10^** | **3.8** | **59** |
|  |  | **6** | **18** | **4** | **38** | **91** | **<10^-10^** | **3.6** | **58** |
|  |  | **16** | **32** | **2** | **-28** | **87** | **<10^-9^** | **3.7** | **61** |
|  |  | **15** | **18** | **-40** | **-2** | **87** | **<10^-9^** | **3.2** | **48** |
|  | **L** | **6** | **-44** | **-6** | **20** | **87** | **<10^-8^** | **3.3** | **45** |
|  | **R** | **8** | **18** | **-18** | **20** | **86** | **<10^-8^** | **3.3** | **41** |
|  | **L** | **5** | **-32** | **-50** | **-4** | **86** | **<10^-8^** | **3.2** | **53** |
|  | **R** | **5** | **22** | **2** | **26** | **86** | **<10^-8^** | **3.2** | **45** |
| **Caudate ncl.** | **L** | **12** | **-34** | **-16** | **-12** | **86** | **<10^-7^** | **4.5** | **83** |
| **White matter** | **R** | **18** | **24** | **20** | **24** | **86** | **<10^-7^** | **3.5** | **57** |
|  | **L** | **36** | **-30** | **-4** | **-26** | **85** | **<10^-7^** | **3.6** | **61** |
|  |  | **6** | **-46** | **2** | **22** | **84** | **<10^-7^** | **2.9** | **36** |
|  | **R** | **7** | **34** | **-60** | **2** | **84** | **<10^-7^** | **4.0** | **71** |
|  |  | **9** | **26** | **-34** | **20** | **84** | **<10^-7^** | **3.9** | **64** |
|  | **L** | **7** | **-20** | **40** | **0** | **83** | **<10^-7^** | **3.9** | **70** |
|  | **R** | **6** | **28** | **-22** | **20** | **81** | **<10^-6^** | **2.8** | **23** |
| ***NAGM*** |  |  |  |  |  |  |  |  |  |
| **Declive** | **L** | **2** | **-22** | **-70** | **-22** | **84** | **<10^-7^** | **-1.5** | **1** |
| **Pyramis** | **L** | **1** | **-8** | **-74** | **-34** | **82** | **<10^-6^** | **-1.3** | **3** |
| **Lentiform ncl.** | **L** | **3** | **-26** | **-8** | **2** | **82** | **<10^-6^** | **-0.6** | **0** |
|  | **R** | **1** | **22** | **4** | **4** | **82** | **<10^-6^** | **-1.7** | **9** |
| **Fusiform gy.** | **R** | **1** | **36** | **-36** | **-18** | **81** | **<10^-6^** | **1.4** | **15** |
| **Inf. semi-lun. lob.** | **L** | **1** | **-32** | **-70** | **-48** | **81** | **<10^-6^** | **-1.8** | **5** |
| **Tuber** | **R** | **1** | **38** | **-64** | **-40** | **81** | **<10^-6^** | **-1.4** | **0** |
| **Pyramis** | **R** | **1** | **18** | **-72** | **-38** | **81** | **<10^-6^** | **-1.4** | **5** |
| **Nodule** | **R** | **1** | **10** | **-58** | **-38** | **81** | **<10^-6^** | **-1.1** | **0** |
| **Culmen** | **R** | **1** | **34** | **-60** | **-32** | **81** | **<10^-6^** | **-0.4** | **0** |
| **Inf. semi-lunar lobule** | **L** | **1** | **-38** | **-70** | **-48** | **80** | **<10^-5^** | **-1.0** | **1** |
| **Thalamus** | **L** | **1** | **-18** | **-28** | **6** | **80** | **<10^-5^** | **-1.7** | **2** |
| **Claustrum** | **L** | **1** | **-34** | **6** | **2** | **79** | **<10^-5^** | **2.0** | **11** |
|  |  | **1** | **-36** | **-12** | **0** | **79** | **<10^-5^** | **2.8** | **21** |
| **Cerebellar tonsils** | **R** | **1** | **30** | **-56** | **-46** | **79** | **<10^-5^** | **-1.8** | **7** |
| ***NAWM*** |  |  |  |  |  |  |  |  |  |
| **White matter** | **L** | **5** | **-22** | **-42** | **-6** | **91** | **<10^-10^** | **1.6** | **17** |
|  | **R** | **3** | **32** | **-6** | **-14** | **86** | **<10^-8^** | **2.4** | **27** |
|  |  | **1** | **54** | **-32** | **8** | **85** | **<10^-7^** | **1.9** | **11** |
|  |  | **1** | **32** | **-14** | **-14** | **83** | **<10^-7^** | **2.9** | **63** |
|  | **L** | **1** | **-34** | **2** | **2** | **83** | **<10^-7^** | **1.0** | **1** |
|  |  | **1** | **-10** | **-46** | **20** | **83** | **<10^-6^** | **1.9** | **6** |
|  |  | **7** | **-56** | **-12** | **30** | **82** | **<10^-6^** | **1.7** | **8** |
|  |  | **1** | **-16** | **16** | **10** | **81** | **<10^-6^** | **-0.7** | **0** |
|  | **R** | **1** | **34** | **-10** | **-12** | **80** | **<10^-6^** | **2.8** | **39** |
|  | **L** | **1** | **-52** | **-52** | **-4** | **81** | **<10^-6^** | **-0.2** | **0** |
|  | **R** | **1** | **32** | **-10** | **-16** | **80** | **<10^-5^** | **3.3** | **60** |
|  | **L** | **1** | **-54** | **-36** | **14** | **80** | **<10^-5^** | **1.5** | **0** |
|  | **R** | **2** | **32** | **-2** | **2** | **80** | **<10^-5^** | **0.2** | **2** |
|  | **L** | **1** | **-6** | **-22** | **32** | **80** | **<10^-5^** | **1.8** | **11** |
|  |  | **1** | **-16** | **-42** | **-2** | **80** | **<10^-5^** | **2.8** | **33** |
|  | **R** | **1** | **36** | **2** | **0** | **80** | **<10^-5^** | **0.9** | **0** |
|  |  | **1** | **34** | **-32** | **-18** | **79** | **<10^-5^** | **2.2** | **20** |
|  |  | **1** | **18** | **52** | **-10** | **79** | **<10^-5^** | **0.8** | **0** |
|  |  | **1** | **44** | **-34** | **30** | **79** | **<10^-5^** | **2.3** | **0** |
