# Supplementary material for: MRI Pattern Recognition in Multiple Sclerosis Normal-Appearing Brain Areas
Source: PLoS One. 2011 Jun 17;6(6):e21138. doi: 10.1371/journal.pone.0021138 (PMC3117878; doi:10.1371/journal.pone.0021138)
Supplement: Table S2 — Cross-validation results for the mapping of regions with disease indicating information based on data corrected for deformation. H, hemisphere; CS, cluster size, i.e. the number of neighboring significant searchlights; x, y, z, Montreal Neurological Institute coordinate of the center of the searchlight classifier with the peak accuracy; DA(%), decoding accuracy; p, probability of the accuracy according to χ2-distribution. Mn t, mean t-value for the group contrast patients minus controls for voxels underlying (a cluster of) significant searchlight classifiers; Vox*(%), percentage of these voxels showing significant results for the contrast (p = 0.001, uncorrected, no cluster size criterion, two-sided). (DOC) [file pone.0021138.s002.doc]

**Table S2.** Cross-validation results for the mapping of regions with disease indicating information based on data corrected for deformation.

| **Tissue / Region** | **H** | **CS** | **x** | **y** | **z** | **DA (%)** | **p** | **Mn t** | **Vox* (%)** |
| --- | --- | --- | --- | --- | --- | --- | --- | --- | --- |
| ***Lesions*** |  |  |  |  |  |  |  |  |  |
| **White matter** | **R** | **1** | **22** | **38** | **-2** | **83** | **<10^-6^** | **2.9** | **22** |
|  |  | **1** | **18** | **40** | **-2** | **81** | **<10^-6^** | **2.5** | **11** |
|  | **L** | **1** | **-20** | **-28** | **42** | **81** | **<10^-6^** | **2.7** | **13** |
|  | **R** | **1** | **32** | **-54** | **26** | **80** | **<10^-5^** | **3.1** | **35** |
|  |  | **2** | **22** | **-28** | **16** | **79** | **<10^-5^** | **2.1** | **8** |
|  |  | **1** | **32** | **-56** | **30** | **79** | **<10^-5^** | **2.6** | **9** |
|  | **L** | **2** | **-22** | **24** | **14** | **80** | **<10^-5^** | **2.2** | **3** |
|  | **R** | **1** | **30** | **-52** | **24** | **79** | **<10^-5^** | **3.5** | **60** |
| ***NAGM*** |  |  |  |  |  |  |  |  |  |
| **Inf. Semi-Lunar LB** | **L** | **1** | **-26** | **-76** | **-50** | **77** | **<10^-5^** | **-0.3** | **0** |
| Parahippocampal Gy. | R | 1 | 38 | -30 | -18 | 70 | <10^-3^ | 0.6 | 0 |
| Subcallosal Gy. | R | 1 | 16 | 4 | -14 | 70 | <10^-3^ | -0.7 | 0 |
| ***NAWM*** |  |  |  |  |  |  |  |  |  |
| White matter | R | 3 | 36 | -60 | 30 | 71 | <10^-3^ | 0.6 | 0 |
|  | L | 1 | -56 | -12 | 30 | 71 | <10^-3^ | 1.1 | 0 |
