# Supplementary material for: MRI Pattern Recognition in Multiple Sclerosis Normal-Appearing Brain Areas
Source: PLoS One. 2011 Jun 17;6(6):e21138. doi: 10.1371/journal.pone.0021138 (PMC3117878; doi:10.1371/journal.pone.0021138)
Supplement: Material S1 — gives further information regarding data preprocessing and detailed results for each tissue-specific pattern recognition analysis (Lesions, NAGM, and NAWM). (DOC) [file pone.0021138.s003.doc]

**­­Supplementary material**

**Methods and materials**

**Data preprocessing**

We started data preprocessing by correcting the MPRAGE and TIRM images of individual subjects for slow-frequency intensity biases using SPM5 (Wellcome Trust Centre for Neuroimaging, Institute of Neurology, UCL, London UK ­ http://www.fil.ion.ucl.ac.uk/spm). Then, a clinician (CP) inspected the TIRM images of individual subjects for hyperintense lesions. Corresponding areas were selected manually and individual lesion masks were generated using in-house software. Lesion coordinates were selected sensitively including also very small and just moderately hyperintense lesions. This strategy should rule out that any information contained in lesions could affect the identification of diagnostic information in areas of NABT as NABT areas were determined by removal of lesion coordinates and coordinates of cerebrospinal fluid (CSF) from all coordinates (see below). Next, TIRM images and lesion masks of individual subjects were co-registered to the individual MPRAGE images using SPM5. Then, MPRAGE, TIRM, and lesion images of individual subjects were normalized to the standard space of the Montreal Neurological Institute (MNI) brain template using SPM5. Coordinates including lesions were excluded from the normalization. Image interpolation included in the normalization procedure was performed using trilinear interpolation.

In the next step, we determined several group masks corresponding to different tissue types. These masks were determined within the standard space of the MNI brain template. We defined a) brain locations where lesions occurred in at least one across all subjects (group lesion mask) and b) brain locations that exclusively contained NABT across the sample (group NABT mask). This mask was further subdivided into c) areas that exclusively contained NAGM (group NAGM mask) and d) areas that exclusively contained NAWM (group NAWM mask).

In order to define the group lesion mask, the normalized lesion masks of individual subjects were additively merged. Whenever an individual mask indicated the occurrence of a lesion for a coordinate, this coordinate was added to the group lesion mask. Correspondingly, the incidence of lesions could vary substantially across the coordinates in the mask ranging from one patient to all patients. In order to define the group NABT mask, we first removed all coordinates that contained CSF (i.e. voxels that had a higher probability of including CSF as compared to GM and WM according to a segmentation procedure of the normalized MPRAGE images of individual subjects using SPM5) in at least one subject from the SPM5 prior brain mask. In the next step, we expanded the group lesion mask by one voxel in each image direction (x, y, and z) and removed the coordinates contained in this expanded group lesion mask. The expansion step was performed in order to rule out that intensity variance of lesion voxels located at the edge of the group lesion mask was introduced into NABT coordinates by the (trilinear) interpolation step applied during normalization or due to partial voluming effects. The expansion dramatically reduced the areas available for the analyses based on normal-appearing brain tissue as it nearly tripled the removed volume from 226288 mm^3^ (volume of original group lesion mask) to 629608 mm^3^ (volume of expanded group lesion mask). However, it guarantees that our estimates of diagnostic information for normal-appearing areas do not indirectly rely on lesions.

Next, we generated the group NAGM mask by reducing the NABT mask to these coordinates that are also included in the grey matter mask of the WFU pickatlas [22]. Finally, we generated the group NAWM mask by reducing the NABT mask to these coordinates that are also included in the white matter mask of the WFU pickatlas [22]. At this point it is important to mention that this atlas is defined for the space of the Montreal-Neurological Institute brain with a spatial voxel resolution of 2 x 2 x 2 mm. Thus, the atlas maps the tissue having the highest density in a given voxel. It does not guarantee that exclusively one type of tissue is contained in a voxel.

In the following step we conducted a within subject intensity standardization (i.e. z­transformation) of the normalized TIRM images of individual subjects. Therefore, we calculated for each subject the mean and standard deviation of voxel intensities located in the group NABT mask. Then, we subtracted the mean from the raw intensity of each voxel and divided the difference by the standard deviation. The intensity z-transformation was exclusively based on intensity parameters of voxels located in the group NABT mask in order to avoid a group specific global intensity bias that could result from a positive lesion load in patients only. Data resulting from the steps depicted up to this point are referred to as ‘uncorrected data’.

We continued by regressing out the variance contained in tissue intensities of the uncorrected data that could be explained by the deformation procedure applied during spatial normalization. First, we used support vector regression (SVR; LIBSVM; http://www.csie.ntu.edu.tw/wcjlin/libsvm) with a linear kernel in order to predict the tissue intensities of a voxel sampled across all subjects based on the deformation (i.e. shift) parameters in the x, y, and z image direction determined for this voxel and each subject during spatial normalization. The shift parameters were calculated for individual MPRAGE images with the deformations toolbox implemented in SPM5. Then, we subtracted the predicted intensity from the raw intensity for each subject. We decided to use SVR instead of the standard general linear model approach because of its higher robustness towards outliers. This correction step was performed in order to rule out that classification could rely on systematic intensity differences between groups induced by the spatial transformation (e.g. due to correction of thalamic atrophy in patients but not in controls). Data resulting from the steps depicted up to this point are referred to as ‘corrected data’.

Then, we conducted a between-subject z-transformation of the data in order to account for intensity variations e.g. due to coil loadings. In the final preprocessing step the data were restricted to the search space of each analysis defined by the group masks as described above.

**Results**

Table S1 gives a comprehensive overview on informative brain areas containing lesions, NAGM, and NAWM as revealed by the analysis of uncorrected data. Table S2 lists informative brain areas revealed by the analysis of data corrected for deformation confounds.
